# Radial and Spiral Stream Formation in *Proteus mirabilis* Colonies

**DOI:** 10.1371/journal.pcbi.1002332

**Published:** 2011-12-29

**Authors:** Chuan Xue, Elena O. Budrene, Hans G. Othmer

**Affiliations:** 1 Mathematical Biosciences Institute, the Ohio State University, Columbus, Ohio, United States of America; 2 Department of Mathematics, Massachusetts Institute of Technology, Cambridge, Massachussetts, United States of America; 3 School of Mathematics and Digital Technology Center, University of Minnesota, Minneapolis, Minnesota, United States of America; MPI for Dynamics and Self-Organization Göttingen, Germany

## Abstract

The enteric bacterium *Proteus mirabilis*, which is a pathogen that forms biofilms *in vivo*, can swarm over hard surfaces and form a variety of spatial patterns in colonies. Colony formation involves two distinct cell types: swarmer cells that dominate near the surface and the leading edge, and swimmer cells that prefer a less viscous medium, but the mechanisms underlying pattern formation are not understood. New experimental investigations reported here show that swimmer cells in the center of the colony stream inward toward the inoculation site and in the process form many complex patterns, including radial and spiral streams, in addition to previously-reported concentric rings. These new observations suggest that swimmers are motile and that indirect interactions between them are essential in the pattern formation. To explain these observations we develop a hybrid model comprising cell-based and continuum components that incorporates a chemotactic response of swimmers to a chemical they produce. The model predicts that formation of radial streams can be explained as the modulation of the local attractant concentration by the cells, and that the chirality of the spiral streams results from a swimming bias of the cells near the surface of the substrate. The spatial patterns generated from the model are in qualitative agreement with the experimental observations.

## Author Summary

Bacteria frequently colonize surfaces and grow as biofilm communities embedded in a gel-like polysaccharide matrix, and when this occurs on catheters, heart valves and other medical implants, it can lead to serious, hard-to-treat infections. *Proteus mirabilis* is an enteric bacterium that forms biofilms on urinary catheters, but in laboratory experiments it can swarm over hard surfaces and form a variety of spatial patterns. Understanding these patterns is a first step toward understanding biofilm formation, and here we describe new experimental results and mathematical models of pattern formation in *Proteus*. The experiments show that swimmer cells in the center of the colony stream inward toward the inoculation site and in the process form many complex patterns, including radial and spiral streams, in addition to concentric rings. To explain these observations we develop a model that incorporates a chemotactic response of swimmers to a chemical they produce. The model predicts that formation of radial streams can be explained as the modulation of the local attractant concentration by the cells, and that the chirality of the spiral streams can be predicted by incorporating a swimming bias of the cells near the surface of the substrate.

## Introduction

A variety of spatial patterns in growing bacterial colonies are found both in nature and in the lab [1]–[10]. When inoculated on semi-solid agar with succinate or other TCA cycle intermediates, motile *Escherichia coli* cells grow, divide, and self-organize into patterns ranging from outward-moving rings of high cell density to chevron patterns, depending on the initial concentration of the nutrient [1], [2]. When grown or simply placed in static liquids, cells quickly reorganize into networks of high cell density comprised of bands and/or aggregates, following exposure to succinate and other compounds. Chemotactic strains of *Salmonella typhimurium*, a closely-related species, can also form concentric rings and other complex patterns in similar conditions [3], [4], and it has been shown that pattern formation in both species is driven by chemotactic interactions between the cells and a self-produced attractant [1]–[3]. The gram-positive bacterium *Bacillus subtilis* forms patterns ranging from highly branched fractal-like patterns to compact forms, depending on the agar and nutrient concentrations [5], [6], [11]. In all these systems proliferation, metabolism and movement of individual cells, as well as direct and indirect interactions between cells, are involved in the patterning process, but the mutual influences and balances between them that lead to the different types of patterns is difficult to dissect experimentally, and is best explored with a mathematical model. Understanding these balances would advance our understanding of the formation of more complex biofilms and other multicellular assemblies [12].

*Proteus mirabilis* is an enteric gram-negative bacterium that causes urinary tract infections, kidney stones and other diseases [13]–[16]. Pattern formation by *Proteus* was described over 100 years ago [17], and the nature of these patterns has since been discussed in many publications. When grown in a liquid nutrient medium, the dominant phenotype of *P. mirabilis* is a vegetative swimmer cell that is 1–2 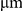 long, has 1–10 flagella and moves using a “run-and-tumble strategy”, similar to that used by *E. coli* [4]. Swimmers respond chemotactically to several amino acids, and can adapt perfectly to external signals [18].

When grown on hard agar *Proteus* forms spectacular patterns of concentric rings or spirals. Swimmers differentiate into highly motile, hyperflagellated, multi-nucleated, non-chemotactic swarmer cells that may be as long as 50–100 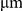, and that move coordinately as “rafts” in the slime they produce [19], [20]. During pattern formation on hard surfaces swarmer cells are found mainly at the leading edge of the colony, while swimmers dominate in the interior of the colony [8], [17], [19], [21]. While much effort has been directed toward understanding the mechanism of swarming, to date little is known about how cells swarm and how cells undergo transitions between swimmers and swarmers [19], [20], [22]–[26], but understanding these processes and how they affect colonization could lead to improved treatments of the diseases caused by *P. mirabilis*.

Traditionally, formation of periodic cell-density patterns in *Proteus* colonies has been interpreted as a result of periodic changes in the velocity of the colony's front, caused by the cyclic process of differentiation and de-differentiation of swimmers into swarmers (see [8]). Douglas and Bisset described in [21] a regime for some strains of *P. mirabilis* in which swarmers form a continuously moving front, while concentric rings of high cell density form well behind that front. This suggests that pattern formation can occur in the absence of cycles of differentiation and de-differentiation. The similarity between this mode of pattern formation and that of *Salmonella* led us to ask whether the underlying mechanism for pattern formation in *P. mirabilis* might also be chemotactic aggregation of the actively moving swimmers behind the colony front.

A number of mathematical models of colony front movement have been proposed, and in all of them swimmer cells are non-motile and swarming motility is described as a degenerate diffusion, in that swarmers only diffuse when their density exceeds a critical value [27]–[31]. The dependence of the front propagation patterns on various parameters in one of these models is given in [29], and while models can reproduce the colony front dynamics, it remains to justify treating the swarming motility as a degenerate diffusion process, since it is likely that the cell-substrate interaction is important. To replicate a periodically propagating front, Ayati showed that swarmers must de-differentiate if and only if they have a certain number of nuclei [30], [31]. It was shown that this may result from diffusion limitations of intracellular chemicals, but biological evidence supporting this assumption is lacking, and further investigation is needed to understand the mechanism of front propagation.

Here we report new experimental results for a continuously-expanding front and show that after a period of growth, swimmer cells in the central part of the colony begin streaming inward and form a number of complex multicellular structures, including radial and spiral streams as well as concentric rings. These observations show that swimmer cells are also motile, and that communication between them may play a crucial role in the formation of the spatial patterns. However, additional questions raised by the new findings include: (1) what induces the inward movement of swimmer cells, (2) why do they move in streams, (3) why do radial streams quickly evolve into spiral streams, and (4) quite surprisingly, why do all the spirals wind counterclockwise (CCW) when viewed from above. To address these questions we developed a hybrid model comprised of a cell-based component for cell dynamics and a continuum component for nutrients and the chemoattractant secreted by swimmer cells. The model has provided biologically-based answers to the questions above and guided new experiments. Previous models, including a continuum chemotaxis model for patterning we developed earlier [32], have limitations discussed later that are not inherent in the hybrid model.

## Results

### Experimental findings

**Figure 1:**
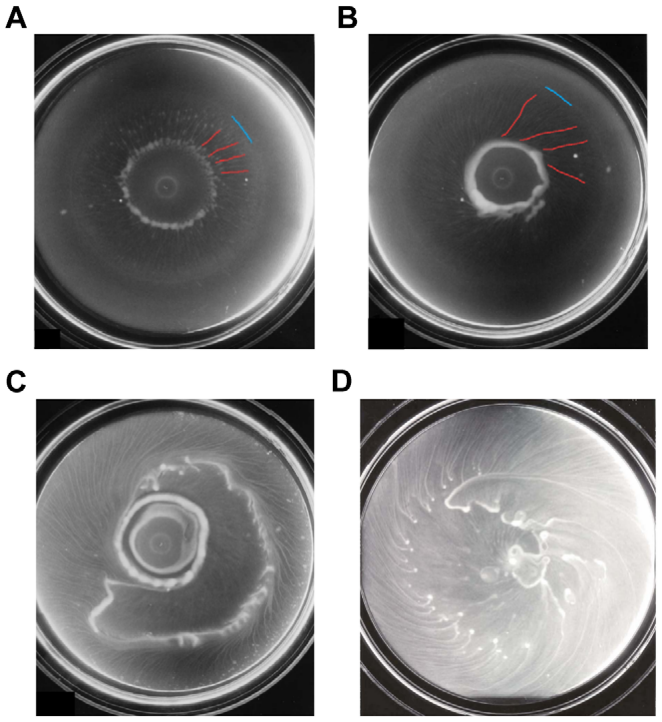
The evolution of a *P. mirabilis* colony. Time after inoculation: (A) 8.5 hours, (B) 9 hours, and (C) 11 hours. (A) initially homogeneous bacterial lawn breaks into radial spokes in the central region of the colony, then bacteria and bacterial aggregates stream inwards following the radial spokes. (B) the radial streams gradually transform into CCW spirals, and the inner ends of each arm join together to form a solid toroidal mass. (C) a second rotating ring forms with spirals that arise further from the center, and a moving train of high cell density forms at some distance from the ring. In (A) and (B), the colony front is highlighted in blue, and a few arms of the streams are highlighted in red. In (C) the colony has covered the entire plate. (D) A different experiment that shows only stream formation without the structure of ring elements.

Previous experimental work focused on expansion of the colony front and neglected the role of movement of swimmers in the pattern formation process in the interior of the colony [8], [19], [21], and the experimental results reported here represent a first step toward understanding their role. After a drop of *P. mirabilis* culture is inoculated on a hard agar-like surface containing rich nutrient, the colony grows and expands. Under the conditions used here, the colony front expands continuously (see Video S1 and Figure S1) - initially as a disc of uniform density. The swarmers exist at the periphery of the colony, and the mean length of the cells decreases towards the center, as observed by others [33]. For the first 5–7 hours, swarmers migrate out the inoculation site, the slime layer gradually builds up and swarmers de-differentiate into swimmer cells behind the leading edge. Later we observe that swimmer cells in the colony stream inward, forming a number of complex patterns (Figure 1). The swimmer population first forms a radial spoke-like pattern in an annular zone on a time scale of minutes, and then cells follow these radial streams inward (1A). The radial streams soon evolve into spirals streams, with aggregates at the inner end of each arm (1B). A characteristic feature of this stage is that the spirals always wind CCW when viewed from above. Different aggregates may merge, forming more complex attracting structures such as rotating rings and traveling trains (1B, C). Eventually the motion stops and these structures freeze and form the stationary elements of the pattern (1B, C). Later, this dynamic process repeats at some distance from the first element of the pattern, and sometimes cells are recruited from that element. In this way, additional elements of the permanent pattern are laid down (1C). On a microscopic level, the transition to the aggregation phase can be recognized as transformation of a monolayer of cells into a complex multi-layered structure. Not every pattern is observable in repeated experiments, (for example, no observable rotating rings can be identified in (1D), probably due to sensitivity to noise in the system and other factors that require further investigation, variations in nutrient availability, etc., but the formation of radial and spiral streams always appear in repeated experiments.

These new findings pose challenges to the existing theories of concentric ring formation in which swimmer cells are believed to be non-motile. Additional questions arise regarding the mechanism(s) underlying the formation of radial and spiral streams, rings and trains by swimmers, and what determines the chirality of the spiral streams. The macroscopic patterns are very different and more dynamic than the patterns formed in *E. coli* or *Salmonella typhimurium* colonies [1]–[3], where cells interact indirectly via a secreted attractant, but the fact that swimmers move up the cell density gradient is quite similar. The non-equilibrium dynamics suggests intercellular communication between individual swimmer cells, and we determined that swimmer cells extracted from these patterns are chemotactic towards several amino acids, including Aspartate, Methionine and Serine (see Table 1). In the following we provide an explanation of the radial and spiral streams using a hybrid cell-based model, by assuming that cells secrete to a chemoattractant that they respond to.

**Table 1:** Chemotaxis analysis of swimmer cells using the amino acid drop assay.

	.1 M	10 mM	1 mM	10 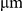	1 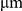
Ala	+	+	−	−	−
Arg	−	−	−	−	−
Asn	−	+	−	−	−
Asp	+	−	+	+	+
Cys	−	−	+	+	−
Glu	+	+	−	−	−
GIn	−	−	−	−	−
Gly	+	+	−	−	−
His	+	+	−	−	−
Ile	−	−	−	−	−
Leu	−	−	−	−	−
Lys	−	−	−	−	−
Met	+	+	−	−	−
Phe	−	+	+	−	−
Pro	−	−	−	−	−
Ser	+	+	+	+	−
Thr	+	−	−	−	−
Trp	−	−	−	−	−
Val	−	−	−	−	−

Method used: *Proteus* cells were collected from the inner area of a growing colony, approximately 1 hr before a projected onset of a streaming phase. Microscopic examination revealed that 90% of cells were 1 to 2 cell length. Cells were resuspended in a minimal growth medium to the OD = .1 to .15 (similar results were obtained with the cells grown in a liquid culture) Drop Assay. 500 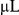 minimal growth medium, 200 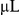 of cell culture (OD = .1 to .15), and 240 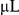 of 1% Methyl cellulose were combined in a 10×35 mm culture dish and mixed until a homogenous state. 4 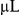 of a respective amino acid solution was added to the center. Cell density distribution in the dish was analyzed after 20–25 minutes. Addition of 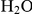 was used as a control. Increase in the cell density in the center indicates that a respective amino acid is an attractant.

### The hybrid cell-based model

**Figure 2:**
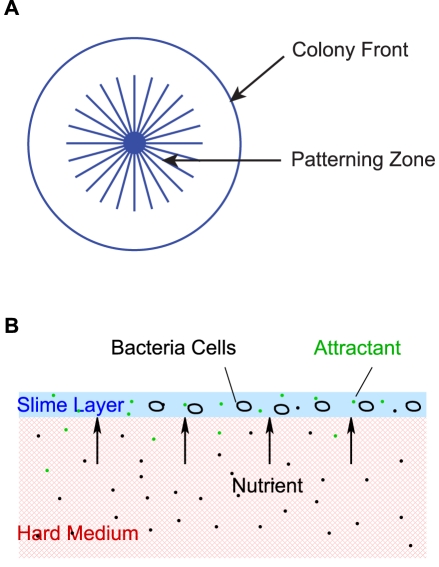
(A) The colony front and the patterning zone. (B) A vertical cross-section of the system. The lower layer is hard agar that contains nutrients, and the top layer is slime generated during colony expansion. Swimmers move in the layer of slime, absorb nutrients that diffuse upward, and secrete attractant. Bacterial flagella are not shown.

The spatial patterns of interest here are formed in the center of the colony where cells are primarily swimmers, while the role of swarmers is mainly to advance the front and to affect the swimmer population by differentiation and de-differentiation. Thus we first focus on modeling the dynamics in the patterning zone in the colony center (Figure 2A), and later we incorporate the colony front as a source of swimmers. This enables us to avoid unnecessary assumptions on the poorly-understood biology of swarming and the transition between the two phenotypes. As noted earlier, swimmer cells are chemotactic to certain factors in the medium, and we assume that they communicate via a chemoattractant that they secrete, and to which they respond. Therefore the minimal mathematical model involves equations for the signal transduction and movement of individual cells, and for the spatio-temporal evolution of the extracellular attractant and the nutrient in the domain shown in Figure 2B. We first focus on understanding the radial and spiral stream formation, which occurs rapidly, and during which the nutrient is not depleted and cells grow exponentially. During this period the nutrient equation is uncoupled from the cell equations and can be ignored. In the radial and spiral streams, cell density is relatively low and cells are still well separated, so we ignore the mechanical interactions between cells.

It has been known for many years that the chemotaxis signal transduction pathway in *P. mirabilis* is very similar to that of *E. coli* [34]–[36]. Recently all the chemotaxis-related genes of *E. coli* have been found in the *Proteus* genome [18], and in view of the genetic similarity between *P. mirabilis* and *E. coli*, we describe motility and signal transduction in the former using the key ideas from the latter.

*E. coli* cells swim using a run-and-tumble strategy, which consists of more-or-less straight runs punctuated by random turns. In the absence of an attractant gradient the result is an unbiased random walk, with mean run time 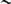
1 s and mean tumble time 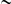
0.1 s. In the presence of an attractant gradient, runs in a favorable direction are prolonged, and by ignoring the tumbling time, which is much shorter than the run time, the movement of each cell can be treated as an independent velocity jump process with a random turning kernel and a turning rate determined by intracellular variables that evolve in response to extracellular signals [37]. The signal transduction pathway for chemotaxis is complex and has been studied extensively both experimentally and mathematically [34]–[36], [38]–[40]. However the main processes are relatively simple, and consist of fast excitation in response to signal changes, followed by adaptation that subtracts out the background signal. These major processes are embedded in the following description of cell behavior.

- Each swimmer cell (with index 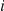) is treated as a point and characterized by its location 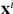, velocity 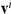, cell-cycle clock 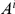 and intracellular variables 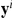.
- Signal transduction in each cell is described by the simple model used in [37], which captures the main features of the signal transduction network. The model involves two variables that evolve according to
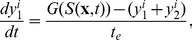
(1)

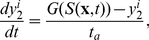
(2)where 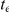, 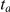 with 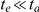 are constants characterizing the excitation and adaptation time scales, 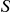 is the local attractant concentration and 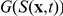 models detection and transduction of the signal. Here 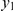 may be identified as the negative of the deviation of 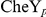 from its steady state, and 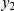 as a measure of the methylation level of the receptors.
- The turning rate and turning kernel of the 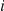-th cell are
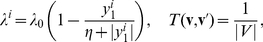
(3)Here 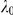 represents the baseline turning rate when there is no external signal gradient, and 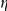 a parameter which indicates how sensitive the turning rate is to the internal variable 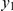. Further, 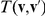 is the turning kernel that appears in the transport equation that describes the velocity jump process [32]: it gives the probability density of turning from 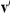 to 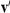 after making the decision to turn. The cell speed 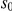 is about 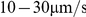 [41], and we assume that it equals 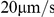. We also assume that there is no directional persistence of cells thus 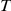 is a constant [42].
- Since the slime layer is very thin, typically 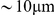, we restrict cell movement to two dimensions.
- Each cell divides every 2 h and is replaced by two identical daughter cells of age 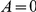.

We assume that cells secrete attractant at a constant rate 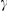 and that it is degraded by a first-order process. Since we neglect cell volume, the attractant is secreted at the center of each cell. The resulting evolution equation for the attractant is
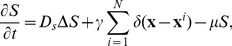
(4)where 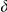 is the Dirac delta function, 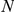 is the total number of cells, and 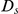 is the diffusion coefficient of the attractant. For simplicity, we also restrict reaction and diffusion of the attractant to two space dimensions, which is justified as follows. Since no attractant is added to the substrate initially, which is much thicker than the slime layer, we assume that the attractant level is always zero in the substrate. We further assume that the flux of the attractant at the interface of the two layers is linear in the difference of its concentration between the two layers. Thus the loss of attractant due to diffusion to the agar can be modeled as a linear degradation, and the degradation constant 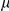 in (4) reflects the intrinsic degradation rate and the flux to the substrate.

In the numerical investigations described below, (4) is solved on a square domain using the ADI method with no-flux boundary conditions, while cells move off-grid. For each time step 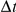 (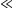 mean run time), (1), (2) are integrated for each cell and the velocity and position are updated by Monte Carlo simulation. Transfer of variables to and from the grid is done using bilinear interpolating operators. A detailed description of the numerical scheme as applied to pattern formation in *E. coli* is given in Appendix A of [32], and for convenience we also included it in the Methods section. After the positions of cells are obtained at each time point, we count the number of cells in each grid and normalize to get the cell density profile in the domain.

### Radial streams result from an instability of the uniform cell distribution

**Figure 3:**
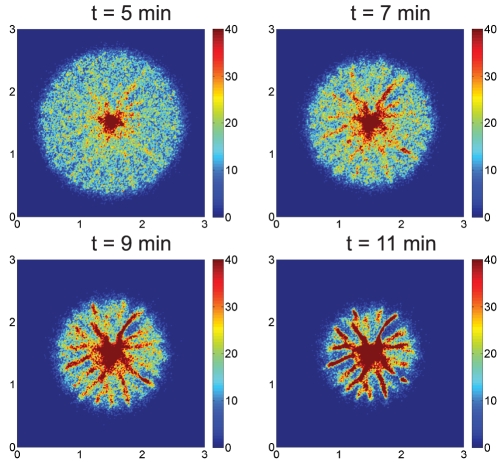
Simulated radial streams. The cell density profile is in unit of 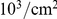. Parameters used: 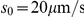, 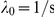, 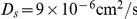, 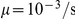, 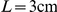, 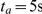, 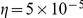, and the secretion rate of the attractant is 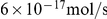 per cell.

Before the emergence of radial streams, the colony expands with a continuous moving front (Video S1 and Figure S1) due to the movement of swarmers, and the cell density is uniform except at the inoculation site, where cells may become non-motile or dormant. During this period of time, the attractant and slime build up and the swarmers de-differentiate to form a population of swimmers. Thus by the end of this period, the attractant concentration can be approximated by a cone-like profile centered at the inoculation site, with a uniform lawn of swimmers laid down. Here we show that starting from this initial condition, the mechanism introduced above can explain radial stream formation on the correct time scale, which is 5–15 minutes. The excitation time scale 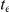 is a fraction of a second, while the adaptation time scale 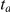 can range from several seconds to several minutes [43], [44]. We assume quasi-steady state for the fast excitation by taking 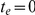 in the numerical investigations below. For simplicity we also assume 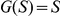, and the intracellular dynamics become
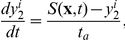


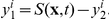
We specify an initial attractant gradient of 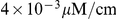 in a disk of radius 1.5 cm, centered at the center of the domain, with zero attractant at the boundary of the disk. For compatibility with later computations on a growing disk, we initially distribute 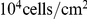 randomly within the disk. (If cells are initially distributed throughout the square domain cells near the four corners, outside the influence of the initial gradient, aggregate into spots, as is observed in *E. coli* as well [32].) Figure 3 shows how this distribution evolves into radial streams that terminate in a high-density region at the center on a time scale of minutes as expected in the experiments. If we double the cell density at the inoculation site, we obtain a qualitatively similar result.

One can understand the breakup into streams as follows. By hypothesis, cells modulate their run lengths in response to the local concentration and the changes they measure via the perceived Lagrangian derivative of attractant along their trajectory, whether or not there is a macroscopic attractant gradient. Small local variations in cell density then lead to local variations in attractant to which the cells respond, and in the absence of a macroscopic gradient, an initially-uniform cell density evolves into a high cell density network, which in turn breaks into aggregates that may then merge (not shown). This has also been found both theoretically and experimentally in *E. coli* (see [1] and Figure 4.4 in [32]). If we describe the cell motion by a 1-D velocity jump process, a linear stability analysis of the corresponding continuum equations predicts that the uniform distribution is unstable, and breaks up into a well-defined spatial pattern (see Figure 4.2, 4.3 in [32]). Numerical solutions of the nonlinear equations confirm this, and experiments in which the grid size is varied show that the results are independent of the grid, given that it is fine enough [32].

**Figure 4:**
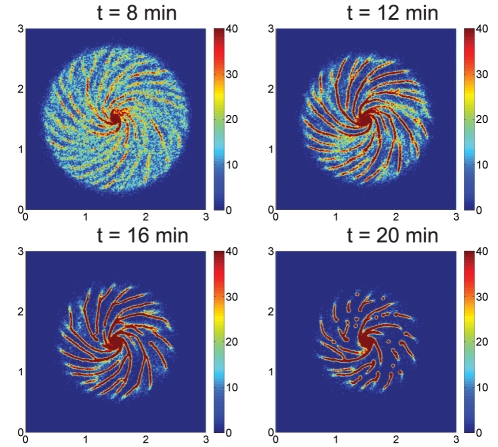
Simulated spiral streams in a disk using a swimming bias of 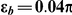. The initial attractant gradient is 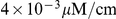, centered as before, and all other parameters are as used for the results in Figure 3.

In the presence of a macroscopic gradient a similar analysis, taken along a 1D circular cross-section of the 2D aggregation field, predicts the breakup of the uniform distribution, but in this situation the 2D pattern of local aggregations is aligned in the direction of the macroscopic gradient. This is demonstrated in a numerical experiment in which cells are placed on a cylindrical surface with constant attractant gradient (Figure S2). Thus the experimentally-observed radial streams shown in Figure 1 and the theoretically-predicted ones shown in Figure 3 can be understood as the result of (i) a linear instability of the uniform cell density, and (ii) the nonlinear evolution of the growing mode, with growth oriented by the initial macroscopic gradient of attractant.

### Spiral streams result from a surface-induced swimming bias

In most experiments the radial streams that arise initially rapidly evolve into spiral streams, and importantly, these spirals always wind CCW when viewed from above. The invariance of the chirality of these spirals indicates that there are other forces that act either on individual cells or on the fluid in the slime layer, and that initial conditions play no significant role. One possible explanation, which we show later can account for the observed chirality, stems from observations of the swimming behavior of *E. coli* in bulk solution and near surfaces. When far from the boundary of a container, *E. coli* executes the standard run and tumble sequence, with more or less straight runs interrupted by a tumbling phase in which a new, essentially random direction is chosen. (There is a slight tendency to continue in the previous direction [41]). However, observations of cell tracks near a surface show that cells exhibit a persistent tendency to swim clockwise (CW) when viewed from above [45]–[47].

Since the cells are small the Reynolds number based on the cell length is very small (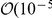)), inertial effects are negligible, and the motion of a cell is both force- and torque-free. Since the flagellar bundle rotates CCW during a run, when viewed from behind, the cell body must rotate CW. When a cell is swimming near a surface, the part of the cell body closer to the surface experiences a greater drag force due to the interaction of the boundary layer surrounding the cell with that at the immobile substrate surface. Suppose that the Cartesian frame has the x and y axes in the substrate plane and that z measures distance into the fluid. When a cell runs parallel to the surface in the y direction and the cell body rotates CW, the cell body experiences a net force in the x direction due to the asymmetry in the drag force. Since the flagellar bundle rotates CCW, a net force with the opposite direction acts on the flagella, and these two forces form a couple that produces the swimming bias of the cell. (Since the entire cell is also torque-free, there is a counteracting viscous couple that opposes the rotation, and there is no angular acceleration.) The closer the cell is to the surface, the smaller is the radius of curvature of its trajectory and the slower the cell speed. Because of the bias, cells that are once near the surface tend to remain near the surface, which increases the possibility of attachment. (In the case of *Proteus* this may facilitate the swimmer-to-swarmer transition, but this is not established.) Resistive force theory has been used to derive quantitative approximations for the radius of curvature as a function of the distance of the cell from the surface and other cell-level dimensions, treating the cell body as a sphere and the flagellar bundle as a single rigid helix [47]. Cell speed has been shown to first increase and then decrease with increasing viscosity of linear-polymer solutions when cells are far from a surface [48], but how viscosity changes the bias close to a surface is not known.

The question we investigate here is whether the microscopic swimming bias of single bacteria can produce the macroscopic spiral stream formation with the correct chirality. We cannot apply the above theory rigorously, since that would involve solving the Stokes problem for each cell, using variable heights from the surface. Instead, we introduce a constant bias of each cell during the runs, *i.e.*,
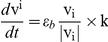
where 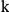 is the normal vector to the surface, and 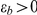 measures the magnitude of the bias in the direction of swimming.

Figure 4 shows the evolution of the cell density using a bias of 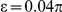, which is chosen so that a cell traverses a complete circle in 50 secs. The simulations show that the initially-uniform cell density evolves into spiral streams after a few minutes and by 12 minutes the majority of the cells have joined one of the spiral arms. The spiral streams persist for some time and eventually break into necklaces of aggregates which actively move towards the center of the domain.

**Figure 5:**
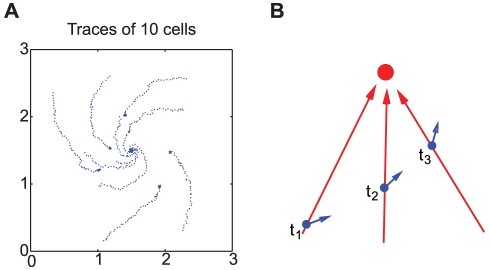
Individual cell tracks and average velocity profile during spiral stream formation in **Figure 4**. (A)The positions of 10 randomly chosen cells, each position recorded every 30 sec by a blue dot. (B) schematics of cell movement with a swimming bias of individual cells.

Figure 5A shows the positions, at 30 second intervals, of 10 randomly chosen cells, and Figure 5B illustrates how to understand the macroscopic chirality based on the swimming bias of individual cells. At 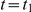 the blue cell detects a signal gradient (red arrow) roughly in the 1 o'clock direction, and on average it swims up the gradient longer than down the gradient. Because of the CW swimming bias, the average drift is in the direction of the blue arrow. At 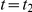 it arrives at the place and ‘realizes’ that the signal gradient is roughly in the 12 o'clock direction, and a similar argument leads to the average net velocity at that spot. As a result of these competing influences, the cell gradually make its way to the source of attractant (the red dot) along a CCW trajectory. Certainly the pitch of the spirals is related to the swimming bias, but we have not determined the precise relationship. The spiral movement has also been explained mathematically for a continuum description of cell dynamics in [32], where the macroscopic chemotaxis equation is derived from the hybrid model in the presence of an external force, under the assumption that the gradient of attractant is shallow. When the swimming bias is constant, the analysis shows that this bias leads to an additional taxis-like flux orthogonal to the signal gradient. However, we show later that the continuum description is not valid for the later stages of patterning in *Proteus*, since attractant gradients become too large.

According to the foregoing explanation, one expects spirals in the opposite direction when experiments are performed with the petri plate upside-down and patterns are viewed from the top, since in this case the relative position of the matrix and slime is inverted and cells are swimming under the surface. This prediction has been confirmed experimentally, and the conclusion is that the interaction between the cell and the liquid-gel surface is the crucial factor that determines the genesis and structure of the spirals.

### Pattern formation on a growing disk

**Figure 6:**
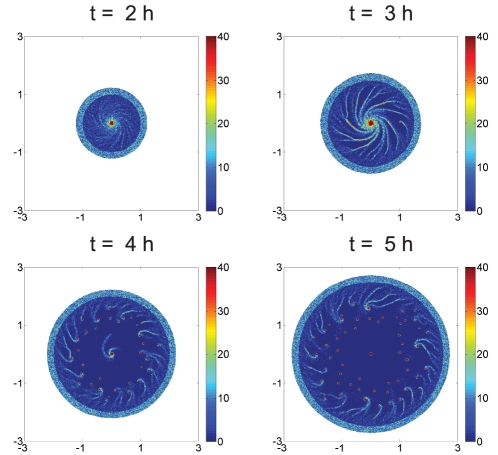
Streams in a growing colony. 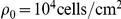
, 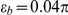, Other parameters used are the same as in Figure 3.

**Figure 7:**
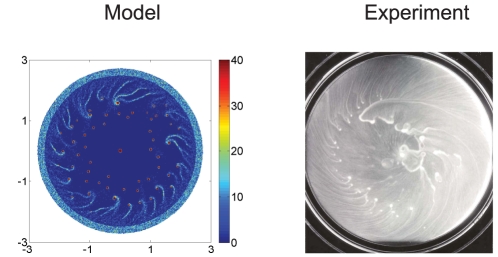
A comparison of predicted and observed spatial patterns. Parameters used are the same as in Figure 3.

From the foregoing simulations we conclude that when the swimming bias is incorporated, the hybrid model correctly predicts the emergence of streams and their evolution into spirals of the correct chirality for experimentally-reasonable initial cell densities and attractant concentration. Next we make a further step toward a complete model by incorporating growth of the patterning domain. The simulation starts when the colony begins to expand. As we indicated earlier, the biology of swimmer/swarmer differentiation and the biophysics of movement at the leading edge are poorly understood. Consequently, we here regard the advancing front as a source of swimmer cells and prescribe a constant expansion rate. Since we simulate from the very beginning of colony expansion, with no attractant in the petri dish, we take 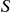 to be zero everywhere as an initial condition. The results of one computational experiment are shown in Figure 6, in which the colony expands outward at a speed of 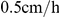, as observed in experiments (Figure S1), and the cells added in this process are swimmer cells. One sees that the early dynamics when the disk is small are similar to the results in Figure 4 on a fixed disk, but as the disk continues to grow the inner structure develops into numerous isolated islands, while the structure near the boundary exhibits the spirals. The juxtaposition in Figure 7 of the numerical simulation of the pattern at 5 hours and the experimental results shown in Figure 1 shows surprisingly good agreement, despite the simplicity of the model. This suggests that the essential mechanisms in the pattern formation have been identified, but others are certainly involved, since the experimental results show additional structure in the center of the disk that the current model does not replicate.

## Discussion

New experimental results reported here show that swimmer cells in the center of the colony stream inward toward the inoculation site, and form a number of complex patterns, including radial and spiral streams in an early stage, and rings and traveling trains in later stages. These experiments suggest that intercellular communication is involved in the spatial pattern formation. The experiments raise many questions, including what induces the inward movement of swimmer cells, why they move in streams, why radial streams quickly evolve into spiral streams, and finally, why all the spirals wind CCW. To address these we developed a hybrid cell-based model in which we describe the chemotactic movement of each cell individually by an independent velocity jump process. We couple this cell-based model of chemotactic movement with reaction-diffusion equations for the nutrient and attractant. To numerically solve the governing equations, a Monte Carlo method is used to simulate the velocity jump process of each cell, and an ADI method is used to solve the reaction-diffusion equations for the extracellular chemicals. The hybrid cell-based model has yielded biologically-based answers to the questions raised by the experimental observations. Starting with an estimate of the attractant level before the onset of the radial streaming as the initial value, we predicted the formation of radial streams as a result of the modulation of the local attractant concentration by the cells. It is observed in *E. coli* that ‘runs’ of single cells curve to the right when cells swim near a surface, and we incorporated this swimming bias by adding a constant angular velocity during runs of each cell. This leads to spiral streams with the same chirality as is observed experimentally. Finally, by incorporating growth of the patterning domain we were able to capture some of the salient features of the global patterns observed.

The streams and spirals reported here share similarities with those formed in *Dictyostelium discoideum*, where cells migrate towards a pacemaker [49]–[52], but there are significant differences. Firstly, the mechanism leading to aggregation is similar, in that in both cases the cells react chemotactically and secrete the attractant. However, since bacteria are small, they do a ‘bakery search’ in deciding how to move - detecting the signal while moving, and constantly modulating their run time in response to changes in the signal. In contrast, *D. discoideum* is large enough that it can measure gradients across it's length and orient and move accordingly [53]. Thus bacteria measure temporal gradients whereas amoeboid cells such as *D. discoideum* measure spatial gradients. In either case the cells respond locally by forming streams and migrate up the gradient of an attractant. However, spirals are less ubiquitous in *D. discoideum*, and when they form they can be of either handedness [54], whereas in *P. mirabilis*, only spirals wound CCW when viewed from above have been observed, which emphasizes the importance of the influence of the cell-substrate interaction when cells swim close to the surface. Experiments in which the patterning occurs in an inverted petri dish lead to spirals with an opposite handedness when viewed from above, which further support our explanation. Our results imply that the spatial patterns observed in *P. mirabilis* can be explained by the chemotactic behavior of swimmer cells, and suggest that differentiation and de-differentiation of the cells at the leading edge does not play a critical role in patterning, but rather serves to expand the colony under appropriate conditions. A future objective is to incorporate a better description of the dynamics at the leading edge when more biological information is available.

The spatial patterns reported here are also different from those observed in other bacteria such as *E. coli* or *Bacillus subtilis*. In the latter, fractal bacterial patterns have been observed [5], [6], and these patterns form primarily at the leading edge of the growing colony. There cell motility plays a lesser role and the limited diffusion of nutrient plays an important role in the pattern formation. In [11], chiral growth patterns have been observed to form at the leading edge of *Paenibacillus* colonies with chirality depending on the concentration of agar in the medium. Those patterns were explained by introducing a phenomenological rotation to the tumbling of cells at the leading edge. However, the spiral streams we presented here form in the center of a growing colony, and the CCW chirality results from the physical property of bacterial swimming when they move close to a surface, namely, a CW individual swimming bias when observed from above [45]–[47].

Further experimental work is needed to validate our primary assumptions and to set the stage for incorporation of more detail into the model. A first step would be to definitively identify the primary attractant and the receptors for it, and to determine whether the primary attractant is also secreted by cells, as assumed here. If several are equally important the mathematical model for individual cells and the equations for the evolution of the attractants would have to be modified, but this poses no new mathematical or conceptual difficulties. Of course if several are involved there are entirely new ways in which the patterns can be influenced by manipulating the attractants. A second set of experiments would be needed to elucidate the behavior of individual cells and determine whether the run-and-tumble description must be modified. This has been done in detail and at great expense for *E. coli*, and would have to be repeated for *Proteus*. The third crucial assumption concerns the mechanism that leads to spirals of fixed chirality. The analysis that leads to our hypothesis for a rotational bias when swimming near a surface relies on the fact that the motion is at low Reynolds number, and therefore, that viscous effects dominate the motion. Accordingly, experiments in which the viscosity is manipulated would shed light on the validity of this assumption, since decreasing the viscosity will decrease the bias and reduce the curvature of the spirals, and conversely for increases in viscosity.

Of course the experimental reality is more complicated than that which our model describes, and this can lead a set of significantly more complex experiments. For instance, the nutrient composition is very complex and nutrient depletion may occur at a later stage, such as during train formation. Further, cells may become non-motile for various reasons, and these factors may play a role in the stabilization of the ring patterns. Another important issue is the hydrodynamic interaction of the swimmer cells with fluid in the slime layer. When cell density is low and cells are well separated we can approximate their movement by independent velocity jump processes plus a swimming bias, but when the cell density is high the cell movement is correlated through the hydrodynamic interactions and this must be taken into account. This hydrodynamic interaction may be an important factor in the formation of the trains observed in experiments.

In previous work the individual cell behavior, including the swimming bias, has been embedded in a continuum chemotaxis equation derived by analyzing the diffusion limit of a transport equation based on the velocity jump process [32]. The resulting equation is based on the assumption that the signal gradient is shallow and the predicted macroscopic velocity in this regime is linear in the signal gradient. A novel feature of the result is that the swimming bias at the individual cell level gives rise to an additional taxis term orthogonal to the signal gradient in this equation. However in the simulations of the patterns presented here we observe steep signal gradients near the core of the patterns and within the streams, and therefore in these regimes the assumptions underlying the continuum chemotaxis model are not valid.

To illustrate the significance of this, we use the function 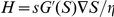 as a measure of the signal gradient detected by a cell, and for each fixed spatial distribution of 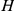, we stochastically simulated the trajectory of 5000 cells with the same initial position and random initial velocity. We found, using least-squares fitting, that the mean, variance, and covariance of the displacement parallel and perpendicular to the gradient can be fit very well by a linear function, and we used these statistics to obtain the macroscopic drift and diffusion rate for each signal gradient 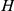 chosen. In the simulations we assumed, without loss of generality, that 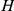 is in the direction of the 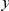-axis, and took the initial positions to be 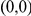. Then we computed the macroscopic drift as
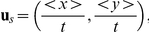
and the diffusion matrix
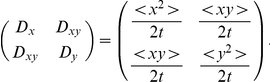



**Figure 8:**
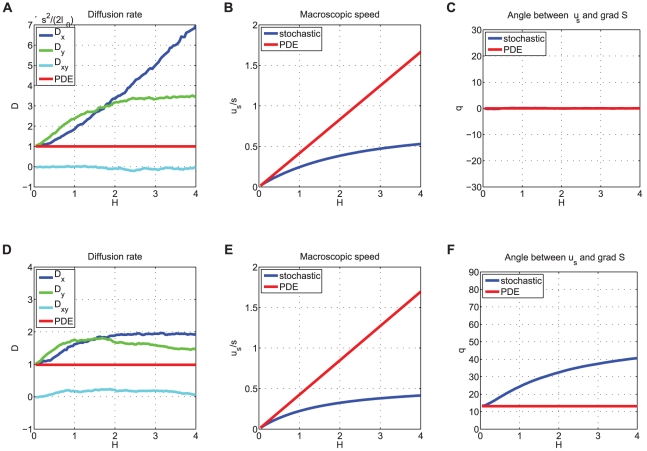
A comparison of the cell-based and the macroscopic predictions of the diffusion matrix 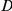, chemotactic velocity 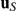, and the angle between 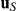 and 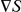. Here 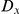 and 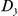 are the diffusion rate perpendicular and in parallel to the signal gradient (along the 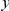-axis), and 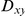 the cross diffusion rate. The horizontal axis (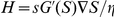) measures the signal gradient interpreted by a cell, with units 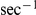. The top row is obtained with no swimming bias as in Figure 3, and the bottom row is obtained with 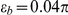 as in Figures 4 and 6. Other parameters used are the same as in the Figures 3, 4, and 6. The blue, green, and cyan curves are obtained from stochastic simulations of the cell-based model, and the red curves are the predictions from the macroscopic chemotactic equation in [32].

Figure 8 compares the statistical results predicted by the cell-based model described above with the formula given in [32], both in the absence of a swimming bias and when there is a bias 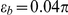, as in Figures 4 and 6. We see that the continuum description given in [32] gives a good approximation for 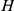 very small (the shallow gradient assumption), but not for 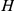 large. The statistical analysis of results from the cell-based model reveals saturation in the macroscopic velocity (Figure 8B, E) and gradient-dependent diffusion coefficients (Figure 8A, D). When there is no bias, both 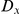 and 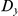 increase with the signal gradient 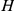 (Figure 8A) and saturate for very large 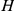 (not shown), while the cross diffusion coefficient 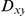 is essentially 0 (Figure 8A). In contrast with this, if there is a swimming bias, the diffusion coefficients 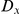 and 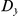 first increase and then decrease before converging to a constant, while 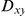 is small but nonzero for intermediate 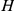 (Figure 8C). These results are very different from the prediction of the continuum model shown in red lines in Figure 8, where the predicted macroscopic velocity exceeds the cell speed in the presence of large signal gradients, and the diffusion of cells is isotropic with a constant coefficient. In addition, statistical analysis of the cell-based model also shows that when there is a swimming bias, the angle between the macroscopic velocity and the signal gradient depends nonlinearly on the magnitude of the signal gradient, in contrast to the prediction from the PDE in [32] (Figure 8F). Thus the hybrid model developed herein successfully describes pattern formation in the presence of large gradients, whereas current continuum descriptions of cell motion do not. Further work is needed to connect the two descriptions in this regime.

## Methods

### Chemotaxis analysis of swimmer cells

To justify the model assumption that swimmer cells are chemotactic to an attractant they produce, we tested if swimmers in the center of the colony have the ability to move chemotacticly. Positive chemotaxis toward each of the common 20 amino acids was tested using the drop assay. Each amino acid was tested at the following concentrations: .1 M, 10 mM, 1 mM, l0 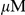, and 1 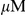 (see Table 1).

Chemotaxis of swimmer cells towards single amino acids was also tested using 0.3% agar plates with different thickness of substrate layer(10 and 20 ml). Each amino acid was used in concentrations varying from 0.25 mM to 7.5 mM in both thicknesses of agar. The plates were point inoculated and placed in a humid chamber at room temperature for at least 20 hrs. Bacteria growing on 10 and 20 ml plates with 0.00l M of Aspartate, Methionine and Serine formed dense moving outer ring which we interpret as a chemotactic ring. Bacteria grown on all remaining amino acids produced colonies with the higher density at the point of inoculation and homogeneous cell distribution in the rest of the colony.

### Numerical algorithm

**Figure 9:**
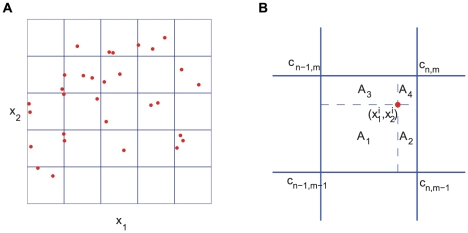
The numerical algorithm for the model. (A) a schematic figure of the domains. The reaction-diffusion equations are solved on the grid, while the cells can move around the whole domain. (B) the area fractions used in defining the interpolators (5, 6).

In the implementation of the cell-based model, cell motion is simulated by a standard Monte Carlo method in the whole domain, while the equations for extracellular chemicals are solved by an alternating direction method on a set of rectangular grid points. In this appendix, we present the numerical algorithm in a two-dimensional domain with only one chemical - the attractant - involved. Each cell is described by its position 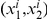, internal variables 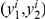, direction of movement 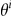 and age 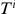 (the superscript 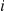 is the index of the cell). Concentration of the attractant is described by a discrete function defined on the grid for the finite difference method (Figure 9A). We denote the time step by 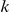, the grid sizes by 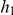 and 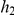.

Since two components of the model live in different spaces, two interpolating operators are needed in the algorithm. 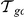 is used to evaluate the attractant concentration that a cell senses. For a cell at 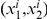, inside the square with vertex indices 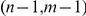, 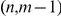, 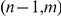 and 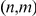, 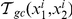 is defined by the bi-linear function:
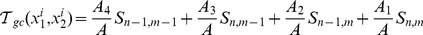
(5)where 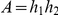 and 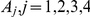 are the area fractions (Figure 9B). On the other hand, the attractant secreted by cells is interpolated as increments at the grid points by 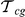. Suppose during one time step 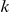, a cell staying at 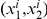 secretes 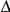 amount of attractant, we then interpolate the increment of the attractant concentration at the neighboring grid points as follows:
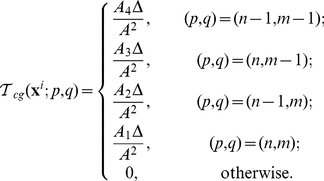
(6)


We consider here a periodic boundary condition. The detailed computing procedure is summarized as follows.

**S1.** Initialization.

- Initialize the chemical fields.
- Initialize the list of swimmer cells. Each cell is put in the domain with random position, moving direction and age. 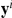 is set to be 0.

**S2.** For time step l (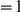 initially), update the data of each cell.

- Determine the direction of movement 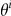 by the turning kernel.
  - Generate a random number 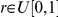;
  - If 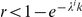, update 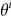 with a new random direction.

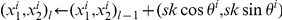
. Apply periodic boundary condition to make sure 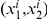 inside the domain,

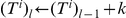
. If 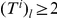 hours, then divide the cell into two daughter cells. This step is only considered when cell growth is considered.
- Update 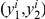 by the equations for the internal dynamics.
  - Determine the attractant concentration before the cell moves 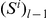 and after the cell moves 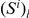 by using the interpolating operator 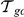.
  - Estimate the attractant level during the movement by 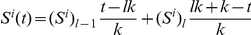 and integrate equation for 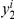 to get 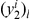.

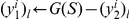
.

**S3.** Compute the source term of the attractant 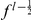 due to the secretion by the cells using the interpolator 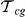


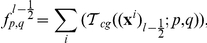
where 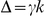.

**S4.** Apply the alternating direction implicit method to the equation of the attractant:
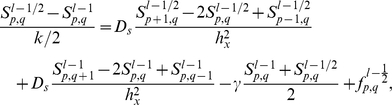


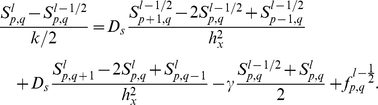
For the boundary grid points, use the periodic scheme.

**S5.** 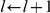. If 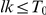, repeat **S2–S4**; otherwise, return.

## Supporting Information

Figure S1 **The radius of the colony as a function of the real time.** The data points here are extracted from the same experiment as Video S1.
(EPS)

Figure S2
**Simulated streams on a 2-D cylindrical surface.** When there is no swimming bias, the alignment of the streams are parallel to the initial attractant gradient. This is demonstrated in the computation where cells are put in a cylindrical surface with constant attractant gradient. The cell density profile is in units of 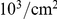, the attractant profile is in unit of 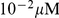. Parameters used are the same as in Figure 3 in the main text.(EPS)

Video S1 **Time evolution of radial and spiral streams.** The real time is shown in the movie. Compressed using Microsoft Video Movie Maker.
(WMV)
